# Dataset on childhood exposure to parenting by lying and its associations with adulthood psychosocial outcomes in a Singapore sample

**DOI:** 10.1016/j.dib.2019.104472

**Published:** 2019-09-03

**Authors:** Peipei Setoh, Siqi Zhao, Rachel Santos, Gail D. Heyman, Kang Lee

**Affiliations:** a Psychology Program, School of Social Sciences, Nanyang Technological University, Singapore; b Dr. Eric Jackman Institute of Child Study, University of Toronto, ON, Canada; c Department of Psychology, University of California, San Diego, USA; d Department of Psychology, Zhejiang Normal University, Jinhua, China

**Keywords:** Lying, Parenting, Dishonesty, Externalizing problems, Internalizing problems, Psychopathy

## Abstract

The present data are reported in the article “Parenting by Lying in Childhood is Associated with Negative Developmental Outcomes in Adulthood” (Setoh et al., in press). Data were collected using online survey. In this dataset, there are 377 responses from young adults from Singapore who reported on their childhood exposure to parenting by lying, their current deceptive behaviors toward parents, and their psychosocial adjustment. Path analysis was performed to better understand parenting by lying - a prevalent, but under-studied parenting practice.

**Table:** Specifications Table

Subject	Psychology
Specific subject area	Developmental and Educational Psychology
Type of data	Tables, figures
How data were acquired	Survey data were acquired using Qualtrics, a web-based survey tool.
Data format	Raw, analyzed
Parameters for data collection	Participants were young adults aged from 18 to 28. Two participants with z-scores beyond three standard deviations of the mean on the lying to parents questionnaire have been removed.
Description of data collection	Participants were recruited from a developmental psychology class and on-campus advertisements. Participants provided their demographic information and completed four online questionnaires.
Data source location	Institution: Nanyang Technological University
	Country: Singapore
Data accessibility	Repository name: DR-NTU
	Direct URL to data: https://doi.org/10.21979/N9/SLNZRW
Related research article	Author's name
	Peipei Setoh, Siqi Zhao, Rachel Santos, Gail D. Heyman, & Kang Lee
	Title
	Parenting by Lying in Childhood is Associated with Negative Developmental Outcomes in Adulthood
	Journal
	Journal of Experimental Child Psychology
	DOI
	In press

**Table:** 

**Value of the Data** • The dataset can be used to understand the associations between retrospective parenting by lying, lying to parents and psychosocial maladjustments amongst emerging adults. • Psychologists and educators who are interested in studying lying behaviors. More specifically, the data provide insights into parenting by lying and its correlates under an eastern, multicultural context. • The data can be examined with different analytic approaches, such as hierarchical linear regression and mediation analysis.

## 1 Data

**Fig. 1:**
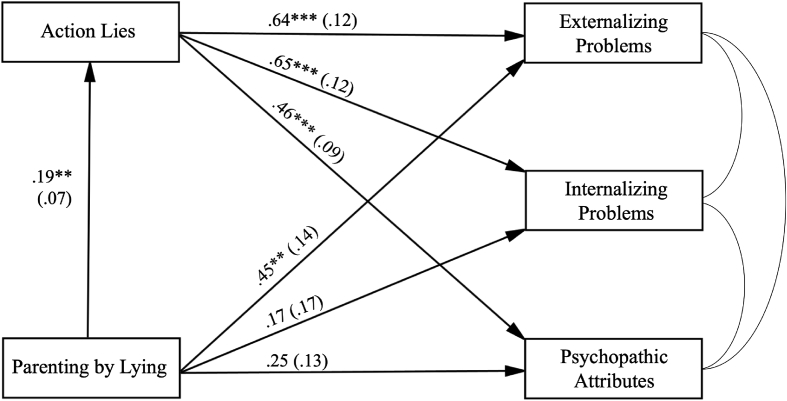
Path analysis with Parenting by Lying as *X*, action lies told to parents as *M*, three maladjustment variables as *Y*s. Gender, age, household income and recruitment mode were included as covariates but are not depicted. Model fit: χ^2^ = 21.099, *df* = 4, *p* = .003; RMSEA = 0.106, 90% CI [0.065, 0.153]; CFI = 0.968; SRMR = 0.035. The coefficients are unstandardized coefficients. *SEs* are in the parentheses. **p < .01; ***p < .001.

**Fig. 2:**
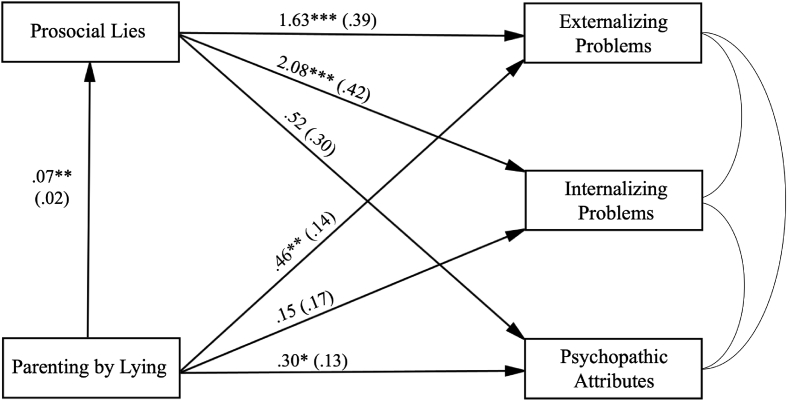
Path analysis with Parenting by Lying as *X*, prosocial lies told to parents as *M*, three maladjustment variables as *Y*s. Gender, age, household income and recruitment mode were included as covariates but are not depicted. Model fit: χ^2^ = 9.467, *df* = 4, *p* = .050; RMSEA = 0.060, 90% CI [0.000, 0.111]; CFI = 0.989; SRMR = 0.019. The coefficients are unstandardized coefficients. *SEs* are in the parentheses. *p < .05; **p < .01; ***p < .001.

**Fig. 3:**
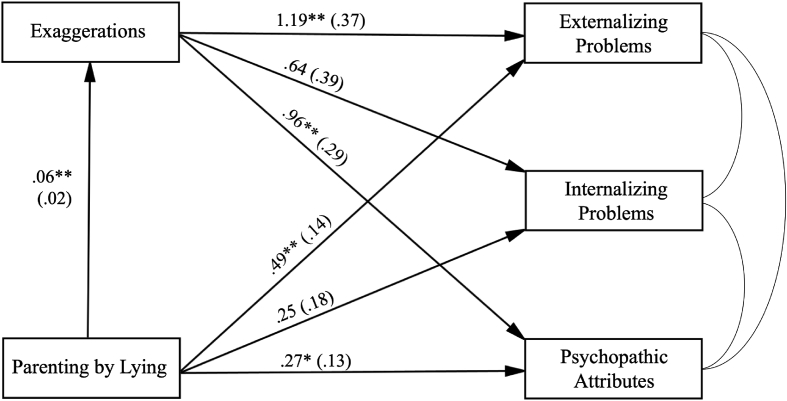
Path analysis with Parenting by Lying as *X*, exaggerations told to parents as *M*, three maladjustment variables as *Y*s. Gender, age, household income and recruitment mode were included as covariates but are not depicted. Model fit: χ^2^ = 2.091, *df* = 4, *p* = .719; RMSEA = 0.000, 90% CI [0.000, 0.057]; CFI = 1.000; SRMR = 0.009. The coefficients are unstandardized coefficients. *SEs* are in the parentheses. *p < .05; **p < .01.

The dataset contains 377 self-report survey responses from a Singapore sample. We measured exposure to parenting by lying in childhood (Table 1), lying behavior toward parents (Table 2) and psychosocial maladjustment amongst young adults [1]. Correlations between the study variables are shown in Table 3, Table 4. Hierarchical linear regression (Table 5) and path analyses (Fig. 1, Fig. 2, Fig. 3) were carried out to understand the factors that associate with parenting by lying.

**Table 1:** Items from the parenting by lying questionnaire.

Category	Male (N = 191)	Female (N = 186)
**Eating**	1.38 (1.07)	1.56 (1.02)
“You need to finish all your food or you will get pimples all over your face.”		
“If you swallow a watermelon seed, it will grow into a watermelon in your stomach.”		
“Finish all your food or you'll grow up to be short.”		
“There's no more candy in the house.” (even though there actually is)		
**Leaving/Staying**	1.51 (0.99)	1.45 (0.94)
“If you don't come with me now, I will leave you here by yourself.” (when parent has no intention of doing it)		
“I won't go out while you are taking a nap.” (when parent intends to go out)		
“If you don't follow me, a kidnapper will come to kidnap you while I'm gone.”		
“Daddy is not out having fun. He is at an important business meeting.” (when the father is actually out for fun)		
**Misbehavior**	1.68 (1.15)	1.69 (1.17)
“If you don't behave, I will call the police.”		
“If you lie to someone, your nose will grow longer.”		
“If you don't quiet down and start behaving, the lady over there will be angry with you.” (it is clear that the lady would not care)		
“If you don't behave, we will throw you into the ocean to feed the fish.”		
**Spending money**	1.54 (1.28)	1.42 (1.26)
“We don't have enough money to buy that toy.” (when family has money)		
A child wants to buy a candy and his/her mother says, “There is no candy in this store.” (when it is not true)		
When passing a toy shop, child asks to go in and buy a toy. Parent says, “We will come back to buy toys next time.” (when parent has no intention to do so)		
“I did not bring money with me today. We can come back another day.” (when the parent did have money and has no intention to go back)		

*Note*: Mean scores of the subscales are shown in the table, separated by gender. *SDs* in parentheses.

**Table 2:** Items from the lying to parents questionnaire.

Category	Male (N = 191)	Female (N = 186)
**Activities and actions**	12.42 (4.31)	11.37 (4.86)
Lie about the things that you are engaged in.		
Are not completely honest with your parents.		
Conceal things that are going on at school from them (relationship with teachers, grades).		
Lie about the reasons why you did not meet an agreement with your parents.		
Consciously do not tell your parents the truth when you have a conversation with them.		
Do not tell your parents important things when asked.		
Lie to your parents about what you do with your friends.		
Only tell your parents part of the story when they ask you something.		
**Prosocial lies**	3.97 (1.42)	3.88 (1.40)
Tell a white lie.		
Sometimes do not tell the truth so you do not have to hurt somebody's feelings.		
**Exaggerations**	3.17 (1.45)	3.17 (1.37)
Exaggerate to your parents about the things you experience.		
Picture things better than they actually are.		

*Note*: Mean scores of the subscales are shown in the table, separated by gender. *SDs* in parentheses.

**Table 3:** Bivariate correlations between deception and adulthood outcomes.

Variable	*M*	*SD*	Externalizing	Internalizing	Psychopathy
**Parenting by lying**					
Eating	1.47	1.05	.13*	.08	–.03
Leaving/Staying	1.48	0.97	.17**	.14**	.17**
Misbehavior	1.68	1.16	.20***	.08	.13*
Spending money	1.48	1.27	.08	.01	.10
**Lying to parents**					
Lies about actions	11.90	4.61	.33***	.28***	.27***
Prosocial lies	3.93	1.41	.26***	.27***	.08
Exaggerations	3.17	1.41	.19***	.09	.13*

*Note*: *p < .05, **p < .01, ***p < .001.

**Table 4:** Bivariate correlations between parenting by lying and lying to parents.

	**Lying to parents**	Lies about actions	Prosocial lies	Exaggerations
**Parenting by lying**	.18**	.14**	.16**	.14**
Eating	.09	.05	.13*	.09
Leaving/Staying	.22***	.19***	.16**	.15**
Misbehavior	.17**	.14**	.13*	.13*
Spending money	.07	.05	.09	.07

*Note*: *p < .05, **p < .01, ***p < .001.

**Table 5:** Summary of hierarchical linear regression for each of the psychosocial maladjustment variables.

Psychosocial Maladjustment	IVs	B	*SE* B	*β*	*t*	*p*	95% CI	*r*_part_	*R*^2^	Δ*R*^2^	Δ*F*^2^
Externalizing	**Step 1**										
	Lying to parents	0.59	0.08	0.36	7.35	<.001	[.43, .75]	0.36	0.13	–	54.08
	**Step 2**										
	Parenting by lying	0.40	0.15	0.13	2.71	.01	[.11, .69]	0.13	0.14	0.02	7.36
Internalizing	**Step 1**										
	Lying to parents	0.54	0.09	0.29	5.96	<.001	[.37, .72]	0.29	0.09	–	35.54
	**Step 2**										
	Parenting by lying	0.14	0.17	0.04	0.82	.41	[-.19, .47]	0.04	0.09	0.002	0.68
Psychopathy	**Step 1**										
	Lying to parents	0.40	0.08	0.25	4.93	<.001	[.24, .56]	0.25	0.06	–	24.33
	**Step 2**										
	Parenting by lying	0.23	0.15	0.08	1.55	.12	[-.06, .53]	0.08	0.07	0.01	2.42

*Note*: Positive linear relationships between lying to parents and psychosocial maladjustment were observed across all the three regressions (Step 1). Overall, greater dishonesty toward parents predicted increased maladaptive behaviors and thinking. Moreover, childhood parenting by lying predicted the severity of externalizing problems after controlling for participants' lying behavior toward parents (Step 2). However, exposure to parenting by lying in childhood did not predict internalizing problems or psychopathic attributes (Step 2).

## 2 Experimental design, materials, and methods

All the data were collected online using Qualtrics. The data consist of demographic information, responses from parenting by lying questionnaire [2], lying to parents questionnaire [3], Adult Self-Report questionnaire [4], [5], as well as the Levenson self-report psychopathy scale [6].

The parenting by lying questionnaire is a 16-item questionnaire developed by Heyman and colleagues [2]. Four categories of parental lies were surveyed: (1) lies that involved eating; (2) lies that involved leaving and/or staying; (3) lies related to children's misbehavior; and (4) lies that involved spending money. For each target item, participants were asked to recall if their parents told them the target lie by indicating “yes”, “no”, or “don't remember”. A “yes” response was coded as 1 while “no” and “don't remember were coded as 0. Total parenting by lying score was created by summing the lies recalled with a “yes” response. The items are listed in Table 1.

The lying to parents questionnaire is a 12-item survey assessed the current frequency of participants’ lying to their parents [3]. It consisted of three aspects of lying to parents: (1) explicit lies about activities and actions; (2) prosocial lies and (3) exaggerations about circumstances and events. Participants indicated how frequently they lied to their parents in adulthood on a 5-point Likert scale, ranging from 1 = never to 5 = very often. The items are listed in Table 2.

The Adult Self-Report questionnaire included 126 items assessing adults’ general adaptive functioning, as well as specific psychosocial dysfunctions [4], [5]. We used age- and gender-normed scores generated by the ASR to measure two types of psychosocial maladjustment: externalizing problems (e.g., aggression, rule-breaking and intrusive behaviors) and internalizing problems (e.g., anxious, depressed and withdrawn behaviors).

The Levenson self-report psychopathy scale consisted of 26 items which assessed psychopathic attributes among the noninstitutionalized population [6]. This instrument evaluated both primary (16 items) and secondary (10 items) psychopathic attributes. Participants indicated their endorsement for each item on a 4-point Likert scale, ranging from 1 = disagree strongly to 4 = agree strongly. Six items were reverse coded.
